# Scleredema of Buschke in a paediatric patient: a novel non-invasive diagnostic and monitoring approach using line-field confocal optical coherence tomography and shear wave elastography

**DOI:** 10.1093/rheumatology/keag203

**Published:** 2026-04-16

**Authors:** Piotr Sobolewski, Mateusz Koper, Anna Dudek, Monika Sztupecka-Rutkowska, Anna Wasążnik-Jędras, Małgorzata Kołos, Irena Walecka

**Affiliations:** Dermatology Clinic, National Institute of Medicine of the Ministry of Interior and Administration, Warsaw, Poland; Chair and Clinic of Dermatology and Pediatric Dermatology, Centre of Postgraduate Medical Education, Warsaw, Poland; Dermatology Clinic, National Institute of Medicine of the Ministry of Interior and Administration, Warsaw, Poland; Dermatology Clinic, National Institute of Medicine of the Ministry of Interior and Administration, Warsaw, Poland; Dermatology Clinic, National Institute of Medicine of the Ministry of Interior and Administration, Warsaw, Poland; Pathomorphological Center, National Institute of Medicine of the Ministry of Interior and Administration, Warsaw, Poland; Pathomorphological Center, National Institute of Medicine of the Ministry of Interior and Administration, Warsaw, Poland; Dermatology Clinic, National Institute of Medicine of the Ministry of Interior and Administration, Warsaw, Poland; Chair and Clinic of Dermatology and Pediatric Dermatology, Centre of Postgraduate Medical Education, Warsaw, Poland

Rheumatology key message

- LC-OCT and shear wave elastography provide objective, non-invasive assessment of disease activity and treatment response in paediatric scleredema Buschke.

Dear Editor, We present a paediatric case of scleredema of Buschke with a classic clinical and histopathological presentation, emphasizing diagnostic evaluation and therapeutic management.

Scleredema of Buschke is an uncommon connective tissue disorder characterized by diffuse, non-pitting skin thickening, most commonly affecting the neck, upper trunk and proximal extremities [1]. Histologically, it is defined by thickened collagen bundles separated by mucin deposits within the dermis, while elastic fibres and adnexal structures remain preserved.

Three clinical subtypes have been described: post-infectious scleredema (type I), scleredema associated with monoclonal gammopathy (type II), and scleredema associated with diabetes mellitus (type III) [2, 3]. In children, the post-infectious form is the most frequently reported and is often preceded by streptococcal upper respiratory tract infections [4].

Due to its rarity and clinical overlap with other sclerosing skin disorders, particularly localized scleroderma, scleredema of Buschke remains a diagnostic challenge. Recent advances in non-invasive skin imaging techniques provide new opportunities for both diagnosis and disease monitoring in sclerosing dermatoses. Line-field confocal optical coherence tomography (LC-OCT) is an emerging imaging modality that combines the advantages of reflectance confocal microscopy and optical coherence tomography, enabling real-time, high-resolution visualization of skin architecture in both vertical and horizontal planes [5]. LC-OCT allows detailed assessment of epidermal and dermal structures with near-histological resolution, reaching depths of up to 500 μm [6]. Skin elastography is another non-invasive imaging modality that enables quantitative assessment of tissue stiffness by measuring the mechanical properties of the skin [7]. In inflammatory and fibrotic skin diseases, elastography provides objective data that correlate with the degree of dermal fibrosis and induration observed clinically [8].

## Case presentation

A 6-year-old boy was admitted to the paediatric department on 27 February 2025 due to progressive skin changes. Two weeks prior to admission, he developed pruritic pustular lesions on the hands, nape and umbilical area, which were initially treated with topical methylprednisolone aceponate followed by tacrolimus ointment without significant improvement.

Within 48 h before hospitalization, painless skin induration of the neck and nape developed, accompanied by facial contour widening. The patient had a history of upper respiratory tract infection at the beginning of February 2025, treated with cefuroxime axetil for 7 days. He was afebrile and denied gastrointestinal or respiratory symptoms. His medical history was notable for drug-induced rash after amoxicillin/clavulanate and clarithromycin 1 year earlier.

On physical examination, the patient was in good general condition. Non-pitting skin induration with increased firmness was noted over the occipital region, nape, neck, ears, trunk and upper extremities, while the lower extremities remained soft and elastic. The skin was painless on palpation and did not restrict joint mobility. Additional findings included livedo reticularis on the lower limbs, subtle truncal erythema, and papular lesions with secondary bacterial infection on the hands and nape.

Laboratory investigations revealed mild leucocytosis (white blood count: 12.17 × 10³/µl) with lymphocytic predominance, thrombocytosis (421 × 10³/µl), slightly elevated erythrocyte sedimentation rate (16 mm/h), and elevated antistreptolysin O titres (ASO; 325 IU/ml). Serum IgM levels were increased, while inflammatory markers (CRP, procalcitonin) and metabolic panels were within normal limits. Extensive autoimmune serology, including ANA, anti-Scl-70, anti-centromere and extractable nuclear antigen antibodies, was negative. Infectious screening for HIV, HBV, HCV, *Borrelia burgdorferi* and respiratory viruses was negative.

A skin biopsy from the nape was performed. Histopathological examination showed normal epidermis and marked thickening of collagen bundles throughout the dermis with mucin accumulation between fibres, confirmed by Alcian blue staining. Elastic fibres demonstrated normal distribution on orcein staining, excluding localized scleroderma. A moderate chronic perivascular and periappendageal inflammatory infiltrate with numerous eosinophils was observed. These findings supported the diagnosis of scleredema Buschke.

Despite initial topical and short-term systemic corticosteroid therapy, the disease progressed, prompting readmission in April 2025 due to expansion of indurated areas and inflammatory skin lesions. After multidisciplinary consultation, systemic glucocorticosteroids were initiated and gradually tapered, and methotrexate (10 mg weekly) with folic acid supplementation was introduced.

In the presented case, LC-OCT was performed before and after systemic treatment, demonstrating characteristic dermal alterations consistent with scleredema, including increased dermal thickness, altered collagen organization and reduced dermal transparency. Importantly, follow-up LC-OCT imaging after corticosteroid and methotrexate therapy revealed a clear normalization of dermal architecture, corresponding with clinical softening of the skin. These findings illustrate the potential role of LC-OCT not only as a diagnostic adjunct but also as a valuable tool for monitoring therapeutic response.

**Figure 1:**
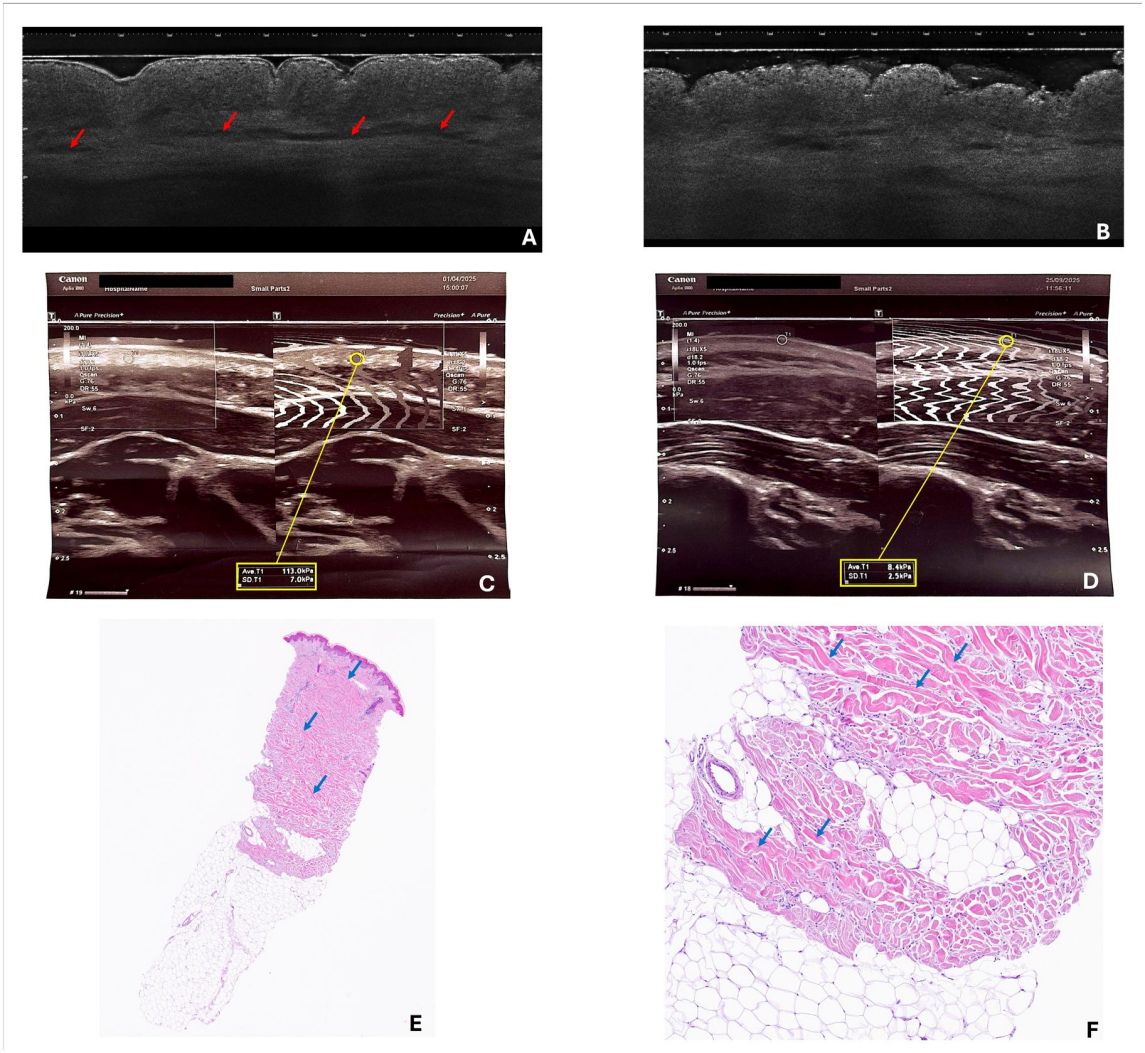
Composite figure illustrating multimodal assessment of scleredema of Buschke: representative line-field confocal optical coherence tomography (LC-OCT), shear wave elastography and histopathology images. (A) LC-OCT image before treatment showing increased dermal thickness and disorganized collagen architecture and elastic fibres (red arrows). (B) LC-OCT image after treatment demonstrating normalization of dermal structure. (C) Shear wave elastography before treatment revealing markedly increased skin stiffness. (D) Shear wave elastography after treatment showing significant reduction in stiffness. (E) Histopathology with normal epidermis and thickened dermal collagen bundles with focal mucin deposition (blue arrows). (F) Histopathology showing thickened collagen fibres extending (blue arrows) toward the subcutaneous fat

Moreover, elastography performed prior to systemic treatment demonstrated markedly increased skin stiffness in clinically affected areas, confirming significant dermal involvement. Control elastographic measurements during follow-up revealed a substantial reduction in skin stiffness after initiation of systemic corticosteroids and methotrexate, which paralleled both clinical improvement and LC-OCT findings. These results underscore the usefulness of elastography as an objective method for monitoring disease activity and treatment response. Representative shear wave elastography, LC-OCT, and histopathology images are provided (Fig. 1). Quantitative elastography results before and after treatment are summarized in Supplementary Table S1.

At follow-up in November 2025, the patient demonstrated significant clinical improvement. The skin of the neck, chest and submental region became soft and elastic, with restored mobility, as confirmed by the ‘pinch test’ for subcutaneous tissue mobility. Residual induration persisted only in the upper arms and forearms. Macroscopic photographs before and during treatment are shown in Supplementary Fig. S1. Laboratory monitoring remained within normal limits.

## Discussion

Scleredema of Buschke is a rare paediatric condition, most commonly presenting as a post-infectious phenomenon following streptococcal infections. Elevated ASO titres and increased IgM levels in this patient support an immune-mediated mechanism triggered by infection.

The differential diagnosis includes localized scleroderma, systemic sclerosis, scleromyxedema and eosinophilic fasciitis. Preservation of elastic fibres, lack of adnexal atrophy and absence of systemic involvement favoured scleredema over other sclerosing dermatoses.

Notably, LC-OCT offers a significant advantage over traditional skin biopsy, particularly in paediatric patients, as it is entirely non-invasive and painless. This is of particular relevance in conditions such as scleredema of Buschke, where repeated assessment may be required to evaluate disease progression or response to treatment. Our observations suggest that LC-OCT may reduce the need for repeat biopsies and support its future application as a complementary imaging technique in the evaluation of paediatric sclerosing skin disorders. Moreover, given its non-invasive nature, reproducibility and ability to provide quantitative data, skin elastography may represent a valuable adjunctive tool in the management of scleredema of Buschke, particularly in paediatric populations. When combined with advanced optical imaging techniques such as LC-OCT, elastography may contribute to a comprehensive, biopsy-sparing diagnostic and follow-up strategy for sclerosing dermatoses.

There is no standardized treatment for scleredema of Buschke. While many paediatric cases resolve spontaneously, progressive or extensive disease may require systemic therapy. Corticosteroids, immunosuppressants such as methotrexate, phototherapy and intravenous immunoglobulins have been reported with variable success [2]. In this case, combined systemic corticosteroids and methotrexate resulted in sustained clinical improvement.

## Conclusions

Scleredema of Buschke should be considered in the differential diagnosis of children presenting with rapidly progressive, painless skin induration following infection. While histopathological examination remains the diagnostic gold standard, this case demonstrates that advanced non-invasive imaging modalities, such as LC-OCT and skin elastography, provide valuable complementary information on dermal structure and tissue stiffness.

LC-OCT enables high-resolution, near-histological visualization of dermal architecture and allows dynamic assessment of treatment response without the need for repeated invasive procedures. Skin elastography offers objective, quantitative evaluation of skin stiffness, closely reflecting disease activity and therapeutic outcomes. Together, these techniques represent a promising, biopsy-sparing diagnostic and monitoring strategy, particularly well suited for paediatric patients.

The integration of LC-OCT and elastography into clinical practice may improve diagnostic accuracy, facilitate longitudinal disease monitoring, and enhance patient comfort. Further studies are warranted to validate their role and establish standardized imaging criteria for scleredema of Buschke and other sclerosing dermatoses.

## Supplementary Material

keag203_Supplementary_Data

## Data Availability

Data are available on request from the corresponding author.
