# Supplementary material for: Scleredema of Buschke in a paediatric patient: a novel non-invasive diagnostic and monitoring approach using line-field confocal optical coherence tomography and shear wave elastography
Source: Rheumatology (Oxford). 2026 Apr 16;65(5):keag203. doi: 10.1093/rheumatology/keag203 (PMC13166886; doi:10.1093/rheumatology/keag203)
Supplement: keag203_Supplementary_Data [file keag203_supplementary_data.docx]

Supplementary material

**Supplementary Table S1. Skin Elastography Measurements Before and After Treatment in the Presented Patient**

| Localization | 01/04/2025 | 25/09/2025 |
| --- | --- | --- |
| Right cheek | 31.0 | 14.1 |
| Right arm | 64.9 | 25.8 |
| Neck | 113.0 | 8.4 |

*Footnote.* Values are expressed as mean skin stiffness and reported in kilopascals (kPa).

**Supplementary Figure S1.**  Macroscopic photographs demonstrating treatment response.

**
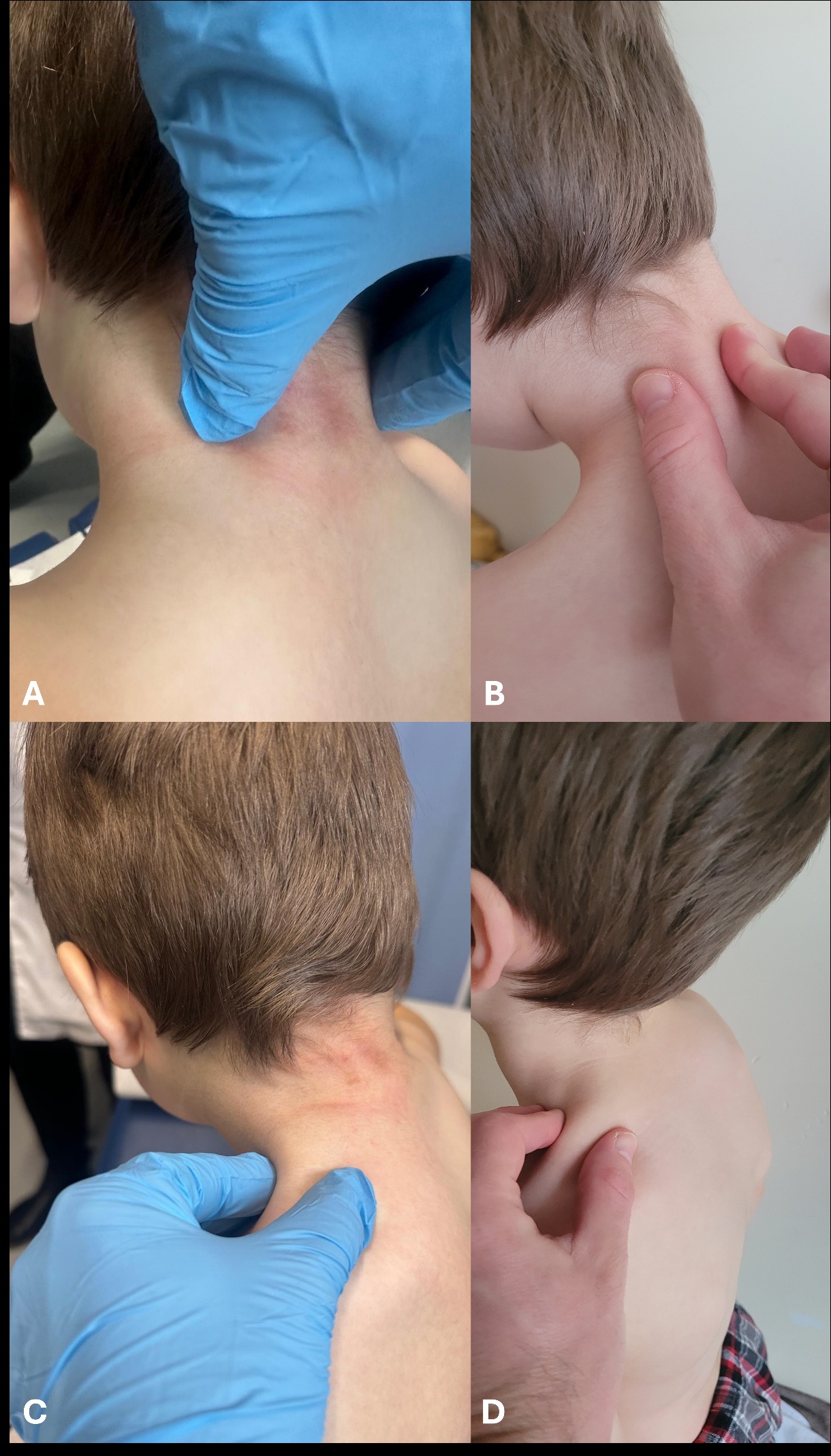
**

*Footnote. (A) pre-treatment photo of the neck showing thickened, indurated skin; (B) pre-treatment photo of the shoulder revealing skin induration; (C) photo of the neck after 6 months of treatment showing softened, elastic skin with improved mobility; (D) photo of the shoulder after 6 months demonstrating significant reduction in induration.*
